# One Health investigation and response to a nationwide outbreak of Rift Valley fever in Rwanda – March to December 2022

**DOI:** 10.1016/j.onehlt.2024.100854

**Published:** 2024-07-04

**Authors:** Leandre Ishema, Soledad Colombe, Fabrice Ndayisenga, Evodie Uwibambe, Eline Van Damme, Marie Meudec, Edson Rwagasore, Denyse Mugwaneza, Wim Van Bortel, Anselme Shyaka

**Affiliations:** a Rwanda Biomedical Centre, Kigali, Rwanda; b Outbreak research team, Department of Public Health, Institute of Tropical Medicine, Antwerp, Belgium; c Rwanda Agriculture and Animal Resources Development Board, Kigali, Rwanda; d Outbreak research team, Department of Biomedical Sciences, Institute of Tropical Medicine, Antwerp, Belgium; e Center for One Health, University of Global Health Equity, Butaro, Rwanda

**Keywords:** Rift Valley fever, Rift Valley fever virus, One health, Rwanda, Transboundary animal diseases, Emerging infectious diseases, Zoonoses, Mosquitoes

## Abstract

Rift Valley fever (RVF) is an emerging zoonotic mosquito-borne disease caused by Rift Valley Fever virus (RVFV), affecting both humans and animals. It is endemic to Rwanda and Tanzania and Uganda which are adjacent countries, with possible transboundary transmissions. Despite the various outbreak reports in Rwanda since 2012, information on the intensity and spread of these outbreaks and their management is scarce. We describe the 2022 outbreak that happened in Rwanda and provide insights into the One Health response implemented during the outbreak.

There were no human cases officially reported. A total of 1339 confirmed RVF animal cases were identified from 21 March until 31 December 2022. The breakdown of the cases per livestock species showed 1285 (96%) cases in cattle, 34 (3%) in goats and 20 (1%) in sheep. Of the confirmed livestock cases, 516 died and 1254 abortions were registered, in all affected species.

The outbreak response was characterized by extensive interventions such as animal spraying with pyrethroid insecticides, vaccinations, and active follow-up of animals and humans in the households with animal cases. In the first phase of the outbreak, animal movements and slaughtering were restricted in the highly affected regions. Gradually, the abattoir slaughter activities were resumed with all animals required to test negative by RT-PCR before slaughter. Remarkably, the public services and hospital laboratories supported both capacity building of veterinary laboratory scientists and testing of animals' samples. The overall response was coordinated by district cross-sectoral teams linking national and community-level actors. Outbreak-related information was synthesized by the district teams and shared at national level while national strategies were communicated to the affected communities through the district structures.

Rwanda's response to RVF provides a proof of concept that multisectoral efforts involving community members in a One Health approach can offer efficient response to zoonotic outbreaks while still protecting the country's economy.

## Highlights

• RVF has been sporadically reported in Rwanda since 2012. Unprecedently, all regions had cases during the 2022 outbreak
• Internal and cross-border livestock movements might play a role in the spread and transmission of RVFV in Rwanda
• Rwanda's RVF response shows multisectoral efforts in a One Health approach offer optimal solutions for zoonoses interventions
• Abattoirs have been used by Rwanda's vet services as effective sentinel sites to detect zoonotic diseases such as RVF
• Surveillance for zoonoses should be strengthened to include small ruminants and livestock that may evade abattoir channels

## 1 Background

Rift Valley fever (RVF) is an emerging zoonotic mosquito-borne disease affecting both humans and livestock [[1], [2], [3]]. RVF mostly occurs in the form of outbreaks, following events of heavy rains, which build an adequate habitat for mosquitoes. Additionally, evidence suggests that there is low-level virus circulation in mosquitoes, animal species (both domestic and wild), and humans during the interepidemic period [4] though the relative role of wildlife in transmission is still not completely known [1]. RVF has historically been endemic in countries along the Rift of East Africa, South Africa, as well as a few countries in West Africa. It is considered a major veterinary and public health emergency, and is increasingly found in countries outside of the known endemic settings [[5], [6], [7]].

Most outbreaks of RVF are initially detected through animal cases, which can reach extremely high numbers through mosquito-to-animal transmission of the virus [1,3]. Human cases often occur through occupational exposure via contact with fluids of infected animals or meat [[1], [2], [3]].

The interepidemic period for RVF has so far been reported as being 5 to 10 years long [1]. Yet recent outbreaks in the East African region have been occurring more frequently, every 1–2 years according to the World Animal Health Information System (WAHIS) [8]. Until 2022, Rwanda itself had been regularly affected by outbreaks of RVF, with known outbreaks reported in animals (cattle, sheep and goats) since 2012 [8] and possibly earlier, and high seroprevalence among livestock already present in the Eastern Province in 2012 [9].

Outbreaks of RVF were reported in cattle and small ruminants from Rwanda in WAHIS every year between 2012 and 2018 except for the year 2015 [8]. Additionally, one publication documents animal cases of RVF in Rwanda in 2020 [10]. All outbreaks up to 2018 had occurred in districts within the Eastern Province of Rwanda only, whereas in 2018, cases were detected in all provinces though not all districts within [11]. The outbreak in 2018 was the largest outbreak prior to 2022 with two human deaths in veterinarians [11]. In May 2018, the first animal cases were detected in the Eastern Province (Kirehe and Ngoma districts). Mass communication, vaccination of herds and and mosquito spraying of animals were put in place in response.

Rwanda continued to be at risk for RVF [12]. In November 2021 and again in February 2022 the Food and Agriculture Organisation (FAO) sent an alert to Rwanda to increase vigilance for RVF [12]. They predicted a risk of RVF occurrence in the East African region, both in animals and humans, in the months of February–April 2022. Persistent hotspots were forecasted in parts of Rwanda, including Western Rwanda [12].

Subsequently, on 21 March 2022, alerts of two cows presenting with possible symptoms of RVF were noticed in the district of Gisagara, Southern Province. Later, ten suspected cows were found between March 23 and 26, 2022, in the Ngoma district of Eastern Province, Rwanda. Consequently, the outbreak of these cases led the Veterinary Services within the Rwanda Agriculture and Animal Resources Development Board to investigate them. RVFV was confirmed by ELISA IgM one week after the initial clinical case. Cross-sectoral task forces were deployed to the affected regions to investigate and respond to the outbreak. The outbreak investigation had two objectives: (i) describing the magnitude of the outbreak, and (ii) understanding the outbreak's source and its spread throughout Rwanda. This report focuses on the investigation and response in the animal sector as mosquito and human testing is still on-going. Although on the decline as of December 2022, animal cases are still being sporadically confirmed and reported, and the Ministry of Agriculture and Animal Resources has not yet declared the outbreak over.

## 2 Methods

### 2.1 Study site and investigation teams

Rwanda is a land-locked country in Eastern Africa divided into five climatic regions: eastern dry and hot plains, temperate central highlands, south-west humid mountain climate, western sea climate around Lake Kivu, and north-western dry-mountain climate [13]. The dry season extends from June to August [14]. In recent years, unusually long dry or rainy seasons have been observed, disrupting the expected seasonality [13,14]. Around 50% of Rwandan households own at least one farm animal. The main types of livestock reared by Rwandan households are cows (28% of the households), followed by goats (19%) and pigs (15%). In 2022, the number of livestock heads per ruminant species were 1.4 million cattle, 1.5 million goats, and 0.3 million sheep [15].

**Fig. 1:**
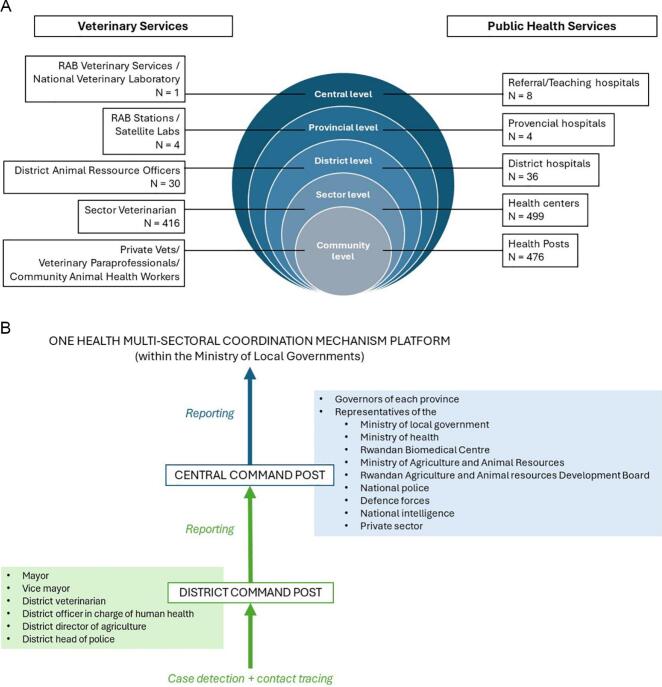
– Organisation of veterinary and human health services in Rwanda for disease surveillance, 2022. **A)** Rwandan administrative levels and veterinary and human health structures in 2022. **B)** Organisation of the One Health command posts during the Rift Valley Fever outbreak, 2022.

Rwanda has a decentralized governance system. The country is geographically split into four provinces plus the City of Kigali. The four provinces and Kigali are subdivided into 30 districts. These districts are divided into 416 Sectors and further into 2148 cells and 14,837 villages. Each village has between 100 and 250 households. Cells and villages constitute the community level (Fig. 1A)

On the side of public health, disease surveillance is coordinated by the Ministry of Health through the Rwanda Biomedical Centre. The surveillance operates from the community level through a vast network of community health workers and a decentralized health care system from health posts to referral hospitals (Fig. 1A).

In veterinary services, disease surveillance is done under the coordination of the Rwanda Agriculture and Animal Resources Development Board (RAB). Due to the lack of veterinary clinics, veterinary services rely on community-based veterinary paraprofessionals, private veterinarians, and community animal health workers. These professionals report to a sector veterinarian (of the administrative entity where a reportable case was found) who cascades the information to the district veterinarian and the later to central level. RAB is also supported by its satellite laboratories, quarantine posts (especially at the borders), and national veterinary laboratory (Fig. 1A). In between outbreaks of animal diseases, surveillance is even-based with any abnormal livestock disease event observed reported by community-based (para-) veterinarians and animal health workers to the Rwanda Agriculture Board via the system described above.

Rwanda adopted a first One Health strategic plan in 2014–2018 [16] and a second One Health Strategic Plan in 2021–2026 [17]. The strategic plans provided a framework for a One Health Multi-Sectoral Coordination Mechanism (OH-MCM) [18] to manage public health challenges at the human-animal-environment interface.

To respond to the 2022 RVF outbreak, the OH-MCM platform activated cross-sectoral task forces. These cross-sectoral task forces, or command posts, were multisectoral teams set-up at district and central level for case detection, contact tracing and reporting (Fig. 1B). The district command posts were composed of the mayor and vice mayor, the veterinarian, the officer in charge of human health, the director of agriculture, and the head of police in the district. The central command post was composed of the governors of each province, representatives of the ministry of local government, the ministry of health, the Rwandan Biomedical Centre, the Ministry of Agriculture and Animal Resources, the Rwandan Agriculture and Animal resources Development Board, the national police, the defence forces, the national intelligence, and finally the private sector (including the meat sector). The central command post reported any outbreak update to the Ministry of Local Governments, where the One Health platform sits [19].

### 2.2 Definitions

We define two phases of the outbreak of 2022: Phase I from March 2022 till May 2022 and Phase II from June till December 2022. Phase I was the initial phase of the investigation during which the response focussed on farms with suspected animals and was characterized by active surveillance. Phase II saw a shift in focus of the investigation and response from farms to slaughterhouses with passive surveillance in place and additional focus on possible human cases.

### 2.3 Data collection

Since human and vector testing is still on-going, we focus for the rest of the methods on animal cases. No active disease surveillance is conducted in wildlife in Rwanda.

Animal case data were collected for the period of 21 March – 31 December 2022. A suspected animal case was defined as any cattle, goat or sheep with fever and one or more of the following: abortion, intra-uterine foetal death, nasal bleeding and/or lacrimation. A confirmed case was defined as any animal with a laboratory confirmation (see below). Daily reporting of cases was done from the community leaders to the district command post via the village, the cell, the sector, the district, and the province*.* The reporting was done via WhatsApp groups. Both aggregated data per district and case-based data were available from the Veterinary Services at the Rwanda Agriculture and Animal Resources Development Board.

During Phase I, suspected animals in farms were tested by the ID Screen® Rift Valley Fever IgM Capture ELISA kits (IDvet, Grabels, France). Testing was done at the National Veterinary Laboratories, and all positive cases were recorded in a line list containing date of diagnostic, district, and outcome of the animal.

During Phase II, all animals presented to slaughterhouses were tested, regardless of symptoms. Laboratory confirmation was done using RealStar® RVFV RT-PCR kit 1.0 RUO (Altona Diagnostics, Hamburg, Germany), which detects three segments (L, M and NSs) and allows minimization of false positives due to prior vaccination. This kit has an analytical sensitivity of 100% if the sample concentration is equal to or higher than 1 copy per μL. The specificity is equally high and no cross-reactivity has been observed with other haemorrhagic fever RNA viruses [20]. Since ELISA IgM was initially used despite prior and on-going vaccination, the rate of false positives by ELISA IgM was assumed to be rather high. By switching from ELISA IgM to PCR testing (which has a much higher specificity and requires active infection), we reduced the number of false positives due to prior vaccination. Testing of animal samples was done at the National Veterinary Laboratories as well as at human provincial hospital laboratories (Musanze, Karongi, Nyagatare, Kigali), under the mandate of the Rwandan Biomedical Centre. Line lists obtained from the National Veterinary Laboratories contained information on all positive cases (district, month of diagnosis). Line lists provided by the Regional Hospitals contained information on all tested cases (district, species, age, sex, pregnancy status and month of diagnosis).

Information on control measures put in place was collected through discussions with involved public health officers and veterinarians.

Denominators for incidence calculation were obtained from Rwanda's latest 2022 census [15].

All cases presented in this manuscript were confirmed.

### 2.4 Data analysis

Analysis of case data was conducted using Excel v2208 and R v4.3 [21]. Categorical variables were summarized using percentages. Epidemiological curves were drawn per month of diagnostic date, which was the earliest date available. Maps were created using QGIS v3.18 and R v4.3 [21]. Monthly maps were created using the line lists while the overall maps were created using overall aggregated data from the Veterinary Services.

## 3 Results

Human and vector data are not available for publication at this stage. The results thus focus on animal cases and on the One Health response put in place.

### 3.1 Epidemiological description of animal cases

**Fig. 2:**
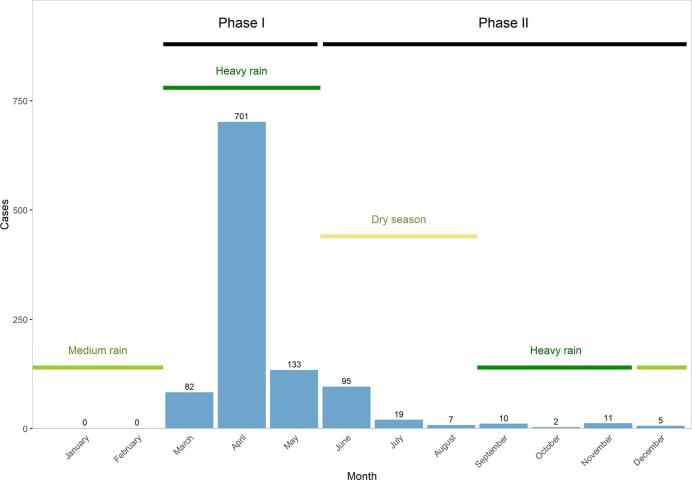
- Number of confirmed cases of RVF by month, Rwanda, January–December 2022.

**Fig. 3:**
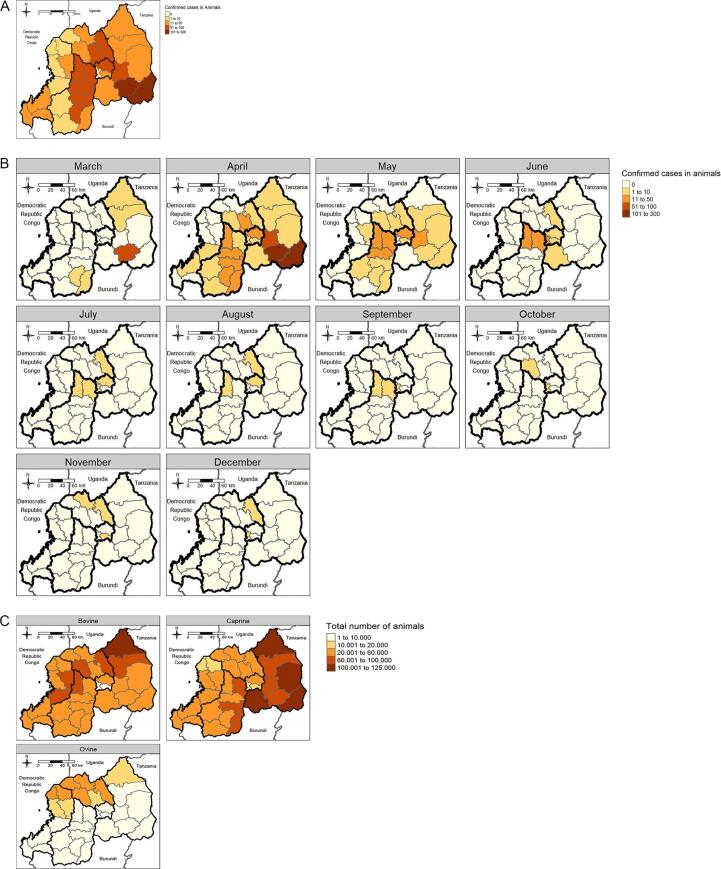
Geographic distribution of confirmed animal cases, RVF outbreak, 2022, Rwanda. **A)** Overall number of RVF confirmed cases (total = 1339) in 2022 in Rwanda, per district (Source: Summary data from Rwanda Agriculture and Animal Resources Development Board, Veterinary Services). **B)** Number of RVF cases confirmed per month (total = 1065) in 2022 in Rwanda per district (Source: line lists from Rwanda Agriculture and Animal Resources Development Board, Veterinary Services). **C**) Number of livestock heads as per the 2022 census in Rwanda per district (Source: National Institute of Statistics of Rwanda).

A total of 1339 confirmed cases of RVF were diagnosed and reported between 31 March 2022 and 31 December 2022 in all districts and provinces of Rwanda (Fig. 2, Fig. 3**A**), with a peak in diagnoses in April 2022 (Fig. 2). Of these 1339 cases, 1285 (96%) were confirmed in cattle, 34 (3%) in goats and 20 (1%) in sheep, representing an incidence of 80 per 100,000 heads in cattle, 2 per 100,000 in goats and 5 per 100,000 in sheep. The overall case fatality rate was 38% (516 deaths). During the same time period, 1254 abortions in animals (all species combined) were reported throughout the country.

Of the 1339 confirmed cases, 1065 (80%) were reported in the line lists, 916 during Phase I (March – May 2022) and 149 during Phase II (June – December 2022). Regional and district data were available for all cases reported in the line lists and are mapped per month of diagnosis in Fig. 3B. Cases were initially detected in three districts in the Eastern Province and two district in the Southern Province, before spreading through the whole country, from the east to the west and from the south to the north. A map of the overall distribution of cattle, goat and sheep heads per district as per the 2022 census [15] is given in Fig. 3C to guide interpretation. The number of confirmed cases per species and per district was not available prior to June 2022 thus preventing the authors from mapping incidence per species and district.

**Fig. 4:**
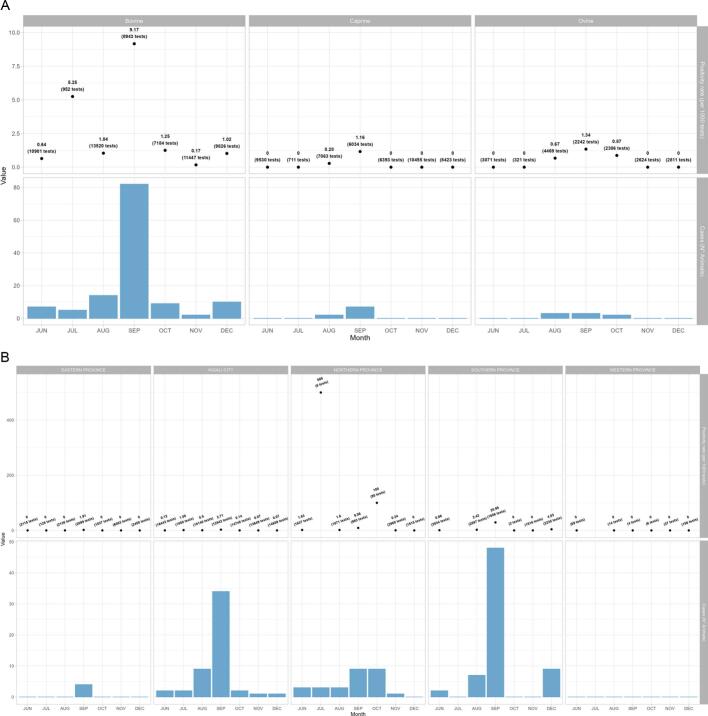
Number of confirmed cases, tests and positivity rate (PR), RVF outbreak, Rwanda, June–December 2022. **A)** Number of confirmed RVF animal cases, tests and positivity rate (PR) by species, June–December 2022, Rwanda. **B)** Number of confirmed RVF animal cases, tests and positivity rate (PR) by region, June–December 2022, Rwanda.

Detailed data on sex, age, and testing results were available from the line list of Phase II. During that Phase, 76/131 (58%) female cases and 39/141 (27%) animals two years of age or younger were confirmed and reported. A total of 129,319 PCR tests were performed during that time, of which 149 (0.12%) were positive. The number of tests conducted per month during Phase II stayed high from June until November 2022 (Fig. 4A and B).

### 3.2 Overall response and control measures

Throughout both Phases (I and II), enhanced surveillance was in place. In addition, control measures were put in place at farm and abattoir level. All herds were sprayed using a pyrethroid insecticide (PERMAPY PLUS © 5 L), by veterinarians, para-veterinarians and community health workers (CHW). In total 105,080 animals were sprayed up to 13 May 2022, representing 13,000 to 79,000 heads being sprayed daily. Typically, farmers would be requested to bring their livestock to designated places where spraying showers had been built, and containment of the animals was possible for vaccination. Alternatively, hand spraying was practised at the designated places.

Intensive vaccination was widely conducted using the Moroccan live attenuated vaccine [22]. The national guidelines recommended that all herds be vaccinated, regardless of distance to a positive herd. Vaccination started in the Eastern Province and was extended to the rest of the country. Zipline drones facilitated delivery, maintaining the cold chain [23]. As of 31 October 2022, 1.3 million cattle (87% of the country's population), 1.4 million goats (93%), and 0.3 million sheep (75%) had been vaccinated.

All testing during phase I was done on samples collected directly at the farms. Any infected animal was given non-specific treatment, including penicillin-streptomycin to prevent secondary bacterial infections, phenylbutazone to reduce fever and vitamin K to stop the bleeding. If the animal died, the body was burned and then burried while animals within the same herd were kept under observation. Trade was wholly interrupted, and slaughterhouses were officially closed to avoid population contamination through meat.

During Phase II, slaughterhouses were re-opened, and all testing and quarantining was done directly at the abattoirs. All animals presented to the abattoir for slaughter were tested and quarantined until the test results were obtained. Any positive test led to isolation of the animal, and supportive treatment with vitamin K until tests returned negative again 14 days after the last positive test. Any negative test led to animal slaughter for trade and consumption. All offends or dead animals were incinerated. Abattoir workers were trained to report suspected cases. The government distributed personal protective equipment (gloves, biohazard bags, masks, gowns, needle vacutainers and vacutainer plastics) to (para-)veterinarians and abattoir workers.

In addition, during phase II, CHW and para-veterinarians were trained in all 500 health centres in Rwanda. Training was focused on clinical signs and symptoms of RVF in humans and animals, transmission dynamics, and surveillance for RVF in Rwanda. Trainees were taught how to report suspected cases promptly and conduct contact tracing. Community leaders also organized workshops where the population learned about the disease and prevention methods. The general population was regularly informed via radiocommunication, social media (Twitter, Facebook), and Rwandan TV.

Contact tracing was conducted throughout the outbreak. Any human contact of known-positive animals was followed up during the incubation period (5 days) and checked daily for malaria-like symptoms. If a contact developed symptoms, they were then tested by PCR. Any positive case would have been brought and quarantined in the district hospital for follow-up and supportive treatment until the return of a negative test.

International borders were never closed, and except for Twitter messages from parastatal institutions' official accounts, no official communication was organized with neighbouring countries.

## 4 Discussion

This report provides an in-depth epidemiological description of an RVF outbreak in Rwanda and describes a successful One Health approach to controlling RVF epidemics.

Though this manuscript could not, at this stage, report results on human cases, there are reports of human cases of Rift Valley detected during this outbreak [24]. The following paragraphs will discuss how joint coordination and collaboration across sectors were witnessed through the task force's actions. These collaborations build transdisciplinary trust and create capacity-sharing opportunities, including for budget and personnel. We argue that the cross-sectoral support in capacity building and medical support for testing livestock samples were significant One Health milestones. This sets a precedent for Rwanda and other resource-limited settings where veterinary services are underfunded and resources are limited.

### 4.1 Spread and transmission of the disease

All RVF outbreaks in Rwanda so far started in the Eastern province and were mostly localized to the Southern and Eastern Provinces. The Eastern Province borders Tanzania to the east and Uganda to the north-east and formal and informal trade routes can be found along these borders, facilitating regular introduction of the virus [[25], [26], [27], [28]]. Additionally, the Eastern Province is home to the Akagera National Park, a large wetland hosting a variety of wildlife species, including buffaloes. Repeated occurrences of RVF in the region could be linked to possible sylvatic cycles involving circulation of RVFV in susceptible wildlife animals and presence of competent vectors in swampy areas [29]. This is supported by high seroprevalence of RVFV in livestock grazing close to the park [9]. The drought followed by flash floods as encountered in Nyagatare District in the Eastern Province from the 3rd to the 5th of March 2022 additionally supports the hypothesis that the outbreak could have started from the hatching of infected mosquito eggs at the start of the heavy rains [1,30]. The Eastern Province is also characterized by an arid climatic condition, making the area permissive for vector-borne RVFV transmission [31,32]. For the affected Southern districts, climatic conditions are less extreme compared to the Eastern Province, but temperature and humidity are equally favourable to the presence of mosquito vectors [32,33]. Both Southern and Eastern provinces also host the highest number of cattle and goats [15]. The repeated occurrence of RVF in the Southern Province may constitute a potential risk for neighbouring Burundi, which had not reported any RVF cases until 10th April 2022 [34].

The Northern Province and, particularly, the Western Province were less affected by the outbreak and reported cases later in the outbreak. Cattle are brought from eastern or southern provinces toward the west and the Democratic Republic of Congo. Consequently, RVF cases in the Northern and Western Provinces might have been due to the spread of the virus through cattle movements. However, we cannot rule out the possibility of RVFV being endemic to Rwanda's Western and Northern Provinces.

While RVF was historically recorded as sporadic in Rwanda, outbreaks have increased in magnitude and frequency with time. This might be linked to the increase in livestock in Rwanda. >60% of the population owned livestock in 2022 [15], and the livestock population has increased by 25.5% over the last 10 years. The increase in livestock heads could lead to an increase in magnitude of RVF outbreaks by introducing a bigger pool of animals susceptible to infection. This increase could also lead to an increase in frequency of outbreaks by encouraging increased animal movements and large animal gatherings. With the country intensifying livestock production, there is a growing need to prevent and optimize control mechanisms for zoonoses.

This increase in magnitude, frequency and spread, might also be linked to ongoing climate changes. The relationship between climate and the epidemiology of Rift Valley Fever has indeed previously been demonstrated, though it is complex. Climate change, particularly through the intensification of El Niño events, leads to increased rainfall and altered rainfall patterns [35,36]. These changes create more favourable conditions for the breeding of mosquitoes, the primary vectors of RVF. As a result, the frequency and severity of RVF outbreaks could increase. In addition, warmer temperatures and changes in rainfall patterns across geographic regions could allow modifications and/or expansion in the geographical spread of RVF infections. Rift Valley Fever transmission is most effective at moderate temperatures around 23 °C–26 °C [37]. More indirectly, changes in water availability might also lead to increased movements and gatherings of animals around rare water sources, leading to increased transmission [38]. Overall, climate change is expected to further exacerbate the risk and impact of RVF outbreaks, necessitating enhanced and adapted surveillance, preparedness, and response efforts.

### 4.2 One Health response and control measures

The outbreak was curbed down quickly after the peak in cases in April 2022, and no resurgence in the number of cases was observed during the second heavy rainy season (September–November 2022), which likely indicates that the response to the outbreak put in place during both Phase I and II what somewhat effective.

Rwanda deployed massive efforts to tackle the outbreak, including large-scale vaccination. Vaccination is a critical element of RVF prevention, and the live attenuated vaccine [29,39] only requires one dose with seemingly high 1-year effectiveness in both cows and small ruminants at low cost. However, vaccination also brings its own set of challenges. Important secondary effects, especially abortion in pregnant animals, have been regularly reported [29,39]. The large number of abortions observed in Rwanda during the outbreak period could be due to wild RVFV infection but also to the extensive vaccination program in place. Logistics are also difficult. The cold chain must be maintained, and to vaccinate so many animals efficiently, farmers are asked to bring their livestock to a vaccination centre in each sector. Multi-dose vials are used with the same syringe and needle at the vaccination centre, possibly participating in RVFV transmission if infectious animals are present. Reassortment between the vaccine strain and wild-types is also possible [40]. The large number of animal cases reported despite mass vaccination has led to concerns regarding the vaccine's effectiveness in Rwanda. Field assessments of vaccine effectiveness in different settings are still missing.

In addition to immunization and insecticide spraying, the country's response was efficient because of the nationwide deployment of command posts, which had previously shown positive performance during COVID-19 [19,41,42]. The command posts' multisectoral composition and coverage allowed them to report any emerging case promptly [42,43]. The command posts also allowed to overcome diagnostic and response challenges that could have affected veterinary services if operating alone. For example, the veterinary services gradually acquired molecular testing capability through assistance from the Rwanda Biomedical Centre. The multi-sectoral collaboration was instrumental in a timely response, sharing testing capacity (reagents and expertise), providing needed personal protective equipment, and supporting awareness campaigns to various community actors.

Community empowerment was key to the outbreak response's efficiency and acceptability. The response focused on the empowerment of abattoir workers and CHW. Educative interventions of abattoir workers were put in place to increase awareness of safer practices needed to protect their health and avoid unintentional release of unsafe meat into the food system. CHWs are embedded in a community that expresses respect and trust toward them [44,45] and can support animal-case reporting and communication with animal owners and herders.

### 4.3 Surveillance and early detection

During phase I of the outbreak, interventions helped identify more active cases and provided initial protection to the animals and public health. The shift to abattoir-based surveillance allowed the resuming of the meat trade and reduced the economic losses usually associated with RVF control measures. Most livestock tested were from the Kigali (Nyabugogo) abattoir, the largest in the country, and slaughtered animals from various Rwandan regions [46]. As previously suggested, slaughterhouses could be a strategic sentinel site to monitor unusual occurrences of zoonotic diseases [[47], [48], [49]]. It offers a sampling framework for RVFV that can be locally implemented and rapidly deployed and, if used routinely, can inform disease trends. As outbreaks of RVF in animals precede human cases, establishing an active animal health surveillance system to detect new cases is essential in providing early warning for veterinary and human public health authorities. This system can be used with participatory maps to improve active livestock surveillance and monitoring [48]. Traders are possibly more likely to bring apparently healthy animals to slaughter to avoid loss associated with animal condemnation [50]; hence, abattoir-based sentinel surveillance would need to be complemented with further investigation and active surveillance at the farm level.

### 4.4 Biases and limitations in the investigation

This investigation needs to be interpreted considering some limitations and biases. Animal health surveillance in Rwanda, including monitoring of animal movements, animal health inspection, and vaccination activities, is highly focused on cows while neglecting small ruminants. In addition, small ruminants are less likely to be slaughtered at abattoirs. The apparent high number of cows infected with RVFV compared to small ruminants in 2022 is probably due to a surveillance and detection bias rather than higher transmission among cows. This is supported by the knowledge that small ruminants are more susceptible to RVFV than cows [3]. The northern part of Rwanda is the main area for small ruminant rearing, so the low number of cases in these provinces may be due to a detection bias. Sick animals are equally less often brought to the abattoir, leading to an underestimation of the number of affected animals [3]. It also means that animal movements toward abattoirs bias the geographical interpretation of the detected cases. The fact that most cases were detected in Kigali (Central) Province, for example, can largely be attributed to the Nyabugogo abattoir being the largest in the country. This, however, doesn't mean that no transmission happened in Kigali. One study done around Nyabugogo abattoir showed the presence of several competent species for RVFV transmission with relatively high proportions of *Culex univitattus*, one of the main vectors of RVFV [51]. The lack of human and vector data does prevent us from triangulating the data and fully understand modes and extent of transmission during this outbreak.

The current outbreak investigation provides an overview of the outbreak in 2022. Further genomic, epidemiological, entomological, and social research is ongoing to clarify the potential introduction routes of the RVFV in Rwanda and the facilitators of the establishment of endemicity. This will help shed more light on the epidemiology of RVF in Rwanda, and it will be a critical finding for mitigating future outbreaks.

## Authors' contributions

LI, SC, WVB and AS led the conception, drafting and finalization of the paper. EVD and EU analysed the data and reviewed the paper. FN, MM, DM, MG, and ER reviewed the paper and participated in drafting discussions. All authors approved the submitted version.

## CRediT authorship contribution statement

**Leandre Ishema:** Conceptualization, Writing – original draft, Writing – review & editing. **Soledad Colombe:** Conceptualization, Data curation, Validation, Writing – original draft, Writing – review & editing. **Fabrice Ndayisenga:** Data curation, Investigation, Supervision, Validation, Writing – review & editing. **Evodie Uwibambe:** Data curation, Validation, Writing – review & editing. **Eline Van Damme:** Visualization, Writing – review & editing, Data curation, Formal analysis. **Marie Meudec:** Writing – original draft, Writing – review & editing. **Edson Rwagasore:** Validation, Writing – review & editing. **Denyse Mugwaneza:** Validation, Writing – review & editing. **Wim Van Bortel:** Conceptualization, Data curation, Formal analysis, Methodology, Project administration, Supervision, Validation, Visualization, Writing – original draft, Writing – review & editing. **Anselme Shyaka:** Conceptualization, Formal analysis, Methodology, Project administration, Supervision, Validation, Writing – original draft, Writing – review & editing, Data curation.

## Declaration of competing interest

The authors report no conflict of interest.

## Data Availability

Data will be made available on request.

## References

[1] Linthicum K.J., Britch S.C., Anyamba A. (2016). Rift Valley fever: an emerging mosquito-borne disease. Annu. Rev. Entomol..

[2] Daubney R. (1932). RIFT VALLEY FEVER. Lancet.

[3] World Animal Health Organization, Chapter 3.1.18 Rift Valley Fever, (n.d.). https://www.woah.org/fileadmin/Home/eng/Health_standards/tahm/3.01.18_RVF.pdf (accessed July 11, 2023).

[4] Sanderson C.E., Jori F., Moolla N., Paweska J.T., Oumer N., Alexander K.A. (2020). Silent circulation of Rift Valley fever in humans, Botswana, 2013–2014. Emerg. Infect. Dis..

[5] Abdo-Salem S., Waret-Szkuta A., Roger F., Olive M., Saeed K., Chevalier V. (2011). Risk assessment of the introduction of Rift Valley fever from the horn of Africa to Yemen via legal trade of small ruminants. Trop. Anim. Health Prod..

[6] El-Harrak M., Martín-Folgar R., Llorente F., Fernández-Pacheco P., Brun A., Figuerola J., Jiménez-Clavero M.Á. (2011). Rift Valley and West Nile virus antibodies in camels, North Africa. Emerg. Infect. Dis..

[7] Carroll S.A., Reynes J.-M., Khristova M.L., Andriamandimby S.F., Rollin P.E., Nichol S.T. (2011). Genetic evidence for Rift Valley fever outbreaks in Madagascar resulting from virus introductions from the east African mainland rather than enzootic maintenance▿. J. Virol..

[8] World Animal Health Organization (2021). World Animal Health Information System (WAHIS).

[9] Umuhoza T., Berkvens D., Gafarasi I., Rukelibuga J., Mushonga B., Biryomumaisho S. (2017). Seroprevalence of Rift Valley fever in cattle along the Akagera-Nyabarongo rivers, Rwanda. J. S. Afr. Vet. Assoc..

[10] Smith L.J., Schurer J.M., Ntakiyisumba E., Shyaka A., Amuguni J.H. (2021). Rift Valley fever knowledge, mitigation strategies and communication preferences among male and female livestock farmers in Eastern Province, Rwanda. PLoS Negl. Trop. Dis..

[11] Dutuze M.F., Ingabire A., Gafarasi I., Uwituze S., Nzayirambaho M., Christofferson R.C. (2020). Identification of Bunyamwera and possible other Orthobunyavirus infections and disease in cattle during a Rift Valley fever outbreak in Rwanda in 2018. Am. J. Trop. Med. Hyg..

[12] Food and Agriculture Organization (2022). https://icpald.org/wp-content/uploads/2022/02/Final-RVF-Alert-February-2022.pdf.

[13] Henninger S.M. (2013). Does the global warming modify the local Rwandan climate?. NS.

[14] World Bank (2022). Climate Knowledge Portal. https://climateknowledgeportal.worldbank.org/country/rwanda/climate-data-historical%23:~:text=Mean%20annual%20temperature%20for%20Rwanda,occurring%20from%20September%20to%20May.

[15] National Institute of Statistics of Rwanda, MAIN INDICATORS: 5th Rwanda Population and Housing Census (PHC) (2023). https://statistics.gov.rw/publication/main_indicators_2022.

[16] Rwanda Ministry of Health (2013). https://rbc.gov.rw/IMG/pdf/one_health.pdf.

[17] Rwanda One Health Steering Committee (2019).

[18] Rwanda Ministry of Health (2021).

[19] Igihozo G., Henley P., Ruckert A., Karangwa C., Habimana R., Manishimwe R., Ishema L., Carabin H., Wiktorowicz M.E., Labonté R. (2022). An environmental scan of one health preparedness and response: the case of the Covid-19 pandemic in Rwanda. One Health Outlook.

[20] Diagnostics Altona (2017). https://altona-diagnostics.com/wp-content/uploads/2023/12/RealStar-RVFV-RT-PCR-Kit-1.0_WEB_CE_EN-S02.pdf.

[21] R Core Team (2023). https://www.R-project.org/.

[22] Boumart Z., Bamouh Z., Hamdi J., Safini N., Tadlaoui K.O., Bettinger G., Watts D.M., Elharrak M. (2020). Safety and immunogenicity of the Rift Valley fever arMP-12 ΔNSm21/384 candidate vaccine in pregnant ewes. Vaccine X.

[23] Griffith E.F., Schurer J.M., Mawindo B., Kwibuka R., Turibyarive T., Amuguni J.H. (2023). The use of drones to deliver Rift Valley fever vaccines in Rwanda: perceptions and recommendations. Vaccines.

[24] De Clerck I. (2023). Outbreak of Rift Valley fever retinitis in Rwanda: novel imaging findings and response to treatment with corticosteroids. Ocul. Immunol. Inflamm..

[25] de Glanville W.A., Allan K.J., Nyarobi J.M., Thomas K.M., Lankester F., Kibona T.J., Claxton J.R., Brennan B., Carter R.W., Crump J.A., Halliday J.E.B., Ladbury G., Mmbaga B.T., Mramba F., Nyasebwa O.M., Rubach M.P., Rostal M.K., Sanka P., Swai E.S., Szemiel A.M., Willett B.J., Cleaveland S. (2022). An outbreak of Rift Valley fever among peri-urban dairy cattle in northern Tanzania. Trans. R. Soc. Trop. Med. Hyg..

[26] Medley A.M., Gasanani J., Nyolimati C.A., McIntyre E., Ward S., Okuyo B., Kabiito D., Bender C., Jafari Z., LaMorde M., Babigumira P.A., Nakiire L., Agwang C., Merrill R., Ndumu D., Doris K. (2020). Preventing the cross-border spread of zoonotic diseases: multisectoral community engagement to characterize animal mobility-Uganda. Zoonoses Public Health.

[27] Sindato C., Karimuribo E.D., Pfeiffer D.U., Mboera L.E.G., Kivaria F., Dautu G., Bernard B., Paweska J.T. (2014). Spatial and temporal pattern of Rift Valley fever outbreaks in Tanzania; 1930 to 2007. PLoS One.

[28] Nyakarahuka L., Whitmer S., Klena J., Balinandi S., Talundzic E., Tumusiime A., Kyondo J., Mulei S., Patel K., Baluku J., Akurut G., Namanya D., Kamugisha K., Cossaboom C., Whitesell A., Telford C., Graziano J., Montgomery J., Nichol S., Lutwama J., Shoemaker T. (2023). Detection of sporadic outbreaks of Rift Valley fever in Uganda through the National Viral Hemorrhagic Fever Surveillance System, 2017–2020. Am. J. Trop. Med. Hyg..

[29] Evans A., Gakuya F., Paweska J., Rostal M., Akoolo L., Van Vuren P.J., Manyibe T., Macharia J.M., Ksizek T.G., Feikin D.D., Breiman R.F., Kariuki Njenga M. (2007). Seroprevalence of Rift Valley fever virus in Kenya wildlife during an inter-epidemic period. Epidemiol. Infect..

[30] Rwanda: Floods - Dec 2019 | ReliefWeb (2021). https://reliefweb.int/disaster/fl-2019-000170-rwa.

[31] Turell M.J. (1989). Effect of environmental temperature on the vector competence of Aedes fowleri for Rift Valley fever virus. Res. Virol..

[32] Seruyange E., Ljungberg K., Muvunyi C., Gahutu J.B., Katare S., Nyamusore J., Gwon Y.-D., Evander M., Norder H., Liljeström P., Bergström T. (2019). Seroreactivity to chikungunya and West Nile viruses in Rwandan blood donors. Vector-Borne and Zoonotic Dis..

[33] Tokash-Peters A.G., Niyonzima J.D., Kayirangwa M., Muhayimana S., Tokash I.W., Jabon J.D., Lopez S.G., Woodhams D.C. (2022).

[34] Njenga G.L., Bett B. (2022). https://www.ilri.org/news/one-health-centre-africa-supports-burundis-response-rift-valley-fever%23:~:text=On%2010%20April%202022%2C%20Burundi,country%20and%20gradually%20spread%20southwards.

[35] Anyamba A., Linthicum K.J., Tucker C.J. (2001). Climate-disease connections: Rift Valley fever in Kenya. Cad. Saude Publica.

[36] Chemison A., Ramstein G., Jones A., Morse A., Caminade C. (2024). Ability of a dynamical climate sensitive disease model to reproduce historical Rift Valley fever outbreaks over Africa. Sci. Rep..

[37] Shocket M.S., Verwillow A.B., Numazu M.G., Slamani H., Cohen J.M., El Moustaid F., Rohr J., Johnson L.R., Mordecai E.A. (2020). Transmission of West Nile and five other temperate mosquito-borne viruses peaks at temperatures between 23°C and 26°C. Elife.

[38] Rust J.M. (2018). The impact of climate change on extensive and intensive livestock production systems. Anim. Front..

[39] Alhaj M. (2016). Safety and efficacy profile of commercial veterinary vaccines against Rift Valley fever: a review study. J Immunol Res.

[40] Grobbelaar A.A., Weyer J., Leman P.A., Kemp A., Paweska J.T., Swanepoel R. (2011). Molecular epidemiology of Rift Valley fever virus. Emerg. Infect. Dis..

[41] Babili A., Nsanzimana S., Rwagasore E., Lester R.T. (2023). SMS-based digital health intervention in Rwanda’s home-based care program for remote management of COVID-19 cases and contacts: a qualitative study of sustainability and scalability. Front Digit Health.

[42] Dzinamarira T., Mapingure M.P., Rwibasira G.N., Mukwenha S., Musuka G. (2021). COVID-19: comparison of the response in Rwanda, South Africa and Zimbabwe. MEDICC Rev.

[43] (2020). Rwanda Ministry of Health, Coronavirus Disease 2019.

[44] LeBan K., Kok M., Perry H.B. (2021). Community health workers at the dawn of a new era: 9. CHWs’ relationships with the health system and communities, Health Research Policy and Systems.

[45] Grace D., Mutua F., Ochungo P., Kruska R., Jones K., Brierley L., Lapar L., Said M., Herrero M., Phuc P.M., Thao N.B., Akuku I., Ogutu F. (2012). Zoonoses Project 4. Report to the UK Department for International Development.

[46] Habarugira G., Mbasinga G., Mushonga B., Chitura T., Kandiwa E., Ojok L. (2016). Pathological findings of condemned bovine liver specimens and associated economic loss at Nyabugogo abattoir, Kigali, Rwanda. Acta Trop..

[47] Falzon L.C., Ogola J.G., Odinga C.O., Naboyshchikov L., Fèvre E.M., Berezowski J. (2021). Electronic data collection to enhance disease surveillance at the slaughterhouse in a smallholder production system. Sci. Rep..

[48] Gerken K.N., Ndenga B.A., Owuor K.O., Winter C.A., Seetah K., LaBeaud A.D. (2022). Leveraging livestock movements to urban slaughterhouses for wide-spread Rift Valley fever virus surveillance in Western Kenya. One Health.

[49] Fèvre E.M., Falzon L.C., Akoko J., Cook E.A.J., Hamilton K.A., Karani M. (2023). Slaughter facilities in East Africa as a focus for one health. One Health Cases.

[50] Clottey St.J.A. (1985). Manual for the Slaughter of Small Ruminants in Developing Countries.

[51] Tantely L.M., Boyer S., Fontenille D. (2015). A review of mosquitoes associated with Rift Valley fever virus in Madagascar. Am. J. Trop. Med. Hyg..

